# Genetic Analysis of Patients with Congenital Hypogonadotropic Hypogonadism: A Case Series

**DOI:** 10.3390/ijms24087428

**Published:** 2023-04-18

**Authors:** Rossella Cannarella, Carmelo Gusmano, Rosita A. Condorelli, Andrea Bernini, Jurgen Kaftalli, Paolo Enrico Maltese, Stefano Paolacci, Astrit Dautaj, Giuseppe Marceddu, Matteo Bertelli, Sandro La Vignera, Aldo E. Calogero

**Affiliations:** 1 Department of Clinical and Experimental Medicine, University of Catania, Via S. Sofia 78, 95123 Catania, Italy; rossella.cannarella@phd.unict.it (R.C.);; 2 Department of Biotechnology, Chemistry and Pharmacy, University of Siena, 53100 Siena, Italy; 3 Diagnostics Unit, MAGI EUREGIO, 39100 Bolzano, Italy; 4 Diagnostics Unit, MAGI’S LAB, 38068 Rovereto, Italy

**Keywords:** hypogonadotropic hypogonadism, Kallmann syndrome, amino acid conservation, molecular modeling

## Abstract

Congenital hypogonadotropic hypogonadism (cHH)/Kallmann syndrome (KS) is a rare genetic disorder with variable penetrance and a complex inheritance pattern. Consequently, it does not always follow Mendelian laws. More recently, digenic and oligogenic transmission has been recognized in 1.5–15% of cases. We report the results of a clinical and genetic investigation of five unrelated patients with cHH/KS analyzed using a customized gene panel. Patients were diagnosed according to the clinical, hormonal, and radiological criteria of the European Consensus Statement. DNA was analyzed using next-generation sequencing with a customized panel that included 31 genes. When available, first-degree relatives of the probands were also analyzed to assess genotype–phenotype segregation. The consequences of the identified variants on gene function were evaluated by analyzing the conservation of amino acids across species and by using molecular modeling. We found one new pathogenic variant of the *CHD7* gene (c.576T>A, p.Tyr1928) and three new variants of unknown significance (VUSs) in *IL17RD* (c.960G>A, p.Met320Ile), *FGF17* (c.208G>A, p.Gly70Arg), and *DUSP6* (c.434T>G, p.Leu145Arg). All were present in the heterozygous state. Previously reported heterozygous variants were also found in the *PROK2* (c.163del, p.Ile55*), *CHD7* (c.c.2750C>T, p.Thr917Met and c.7891C>T, p.Arg2631*), *FLRT3* (c.1106C>T, p.Ala369Val), and *CCDC103* (c.461A>C, p.His154Pro) genes. Molecular modeling, molecular dynamics, and conservation analyses were performed on three out of the nine variants identified in our patients, namely, *FGF17* (p.Gly70Arg), *DUSP6* (p.Leu145Arg), and *CHD7* p.(Thr917Met). Except for *DUSP6*, where the L145R variant was shown to disrupt the interaction between β6 and β3, needed for extracellular signal-regulated kinase 2 (ERK2) binding and recognition, no significant changes were identified between the wild-types and mutants of the other proteins. We found a new pathogenic variant of the *CHD7* gene. The molecular modeling results suggest that the VUS of the *DUSP6* (c.434T>G, p.Leu145Arg) gene may play a role in the pathogenesis of cHH. However, our analysis indicates that it is unlikely that the VUSs for the *IL17RD* (c.960G>A, p.Met320Ile) and *FGF17* (c.208G>A, p.Gly70Arg) genes are involved in the pathogenesis of cHH. Functional studies are needed to confirm this hypothesis.

## 1. Introduction

Congenital hypogonadotropic hypogonadism (cHH) is a rare clinical condition of heterogeneous genetic etiology. It can affect both male and female patients and presents with signs/symptoms of hypogonadism alone or associated with other clinical manifestations. The latter include skeletal malformations, a cleft palate or lip, dental agenesis, kidney agenesis, ear malformations, variable congenital deafness, ataxia, and hyposmia/anosmia. The disease is called Kallmann syndrome (KS) when smell impairment is present.

CHH affects 1:4000 newborns, with a higher prevalence in males than in females (3–5:1) [1,2]. It is characterized by isolated low serum levels of sex steroid hormones in the presence of low or inappropriately normal serum gonadotropins, whereas the remaining hypothalamic–pituitary function is normal.

CHH/KS is a complex genetic disease. More than 30 genes have been involved in its pathogenesis until now, including the most up-to-date list of 46 genes [3]. However, due to the widespread use of next-generation sequencing (NGS) technology, this list is likely to further grow. The gene mechanisms involved in the pathogenesis of cHH/KS influence the fine regulation of the migration and/or function of gonadotropin-releasing hormone (GnRH) neurons [4]. In particular, these alterations can affect GnRH neuron differentiation, migration and axon projection, GnRH neuron homeostasis, or GnRH function. However, the role of some genes is still unclear [5] (Supplementary Table S1).

*KAL1* (also named *ANOS1*) was the first-described gene leading to the identification of cHH/KS [6,7]. It encodes for anosmin-1 and restrictedly modulates interstitial matrix differentiation in neuroblasts [8,9]. Since then, several other genes have been identified. Indeed, various and numerous proteins are involved in the delicate and complex mechanism of GnRH neuron function, such as kisspeptin (KISS1) and its receptor (*KISS1R*), whose variants cause isolated cHH [10,11], or *chromodomain DNA-binding helicase protein* (*CHD7*), whose variants lead to cHH with or without CHARGE syndrome [12].

cHH/KSS has a complex inheritance mechanism, which does not always follow the rules of Mendelian inheritance. The *PROKR2* gene, for example, was discovered to be involved in the pathogenesis of KS in 2006, when a homozygous knock-out mouse model with cHH due to GnRH deficiency and hypotrophy of the olfactory bulbs was published [13]. In the same year, mutations of the *PROKR2* gene were reported in patients with KS [14]. Subsequently, variations of this gene have been described in homozygous, heterozygous, or compound heterozygous states [15]. Furthermore, since cHH occurred in patients with loss-of-function *PROK2* gene mutations, autosomal recessive transmission was postulated [16]. Subsequently, the presence of heterozygous *PROKR2* mutations in unaffected first-degree relatives of patients with cHH/KS led to speculation on the occurrence of digenic/oligogenic transmission [17,18,19]. This mode of transmission appears to account for 2.5–15% of all patients with cHH [20].

We report a clinical and genetic investigation of five unrelated patients with cHH/KS analyzed using a customized genetic panel. The consequences of the identified variants on gene function were evaluated by analyzing amino acid conservation across species and by using molecular modeling.

## 2. Patients and Methods

### 2.1. Patients

In this study, patients of the Division of Endocrinology, Metabolic Diseases and Nutrition, University-Teaching Hospital Policlinico “San Marco—G. Rodolico”, University of Catania (Catania, Italy), with a suspicion of cHH/KS were considered for inclusion. Only those who met the diagnostic criteria set by the European Consensus Statement document [4] were finally included. These are divided into clinical, hormonal, and radiological criteria. In detail, the clinical criteria include the absence or incomplete development of sexual characteristics (testicular volume < 4 mL at age 14 in males; Tanner stage 1 breasts at age 13 or primary amenorrhea at the age of 15 in females) and an absent or insufficient growth spurt. The hormonal criteria consist of low levels of sex hormones and gonadotropins in basal conditions and after stimulation with GnRH [4]. Finally, only patients with a normal morphological appearance of the hypothalamic–pituitary regions on magnetic resonance imaging (MRI) scans were considered for inclusion.

Patients with CDGP or systemic (hemochromatosis, sarcoidosis, histiocytosis, and thalassemia)/acquired (pituitary adenomas and/or brain tumors; Rathke’s cleft cyst; pituitary apoplexy; radiation of the brain or pituitary; and treatments, including drugs, such as steroids, opiates, or chemotherapy) causes of hypopituitarism/hypogonadism were excluded [4]. However, patients with functional causes of hypogonadism (malnutrition, eating disorders, and excessive exercise) were included to assess whether their genetic background could interfere with the function of the hypothalamic–pituitary–gonadal axis in the presence of disturbing external factors.

Five unrelated Caucasian patients (four males and one female) met the inclusion criteria and agreed to be included. They underwent blood sampling for a genetic analysis.

### 2.2. Genetic Analysis

DNA was extracted from peripheral blood using a commercial kit (Samag Blood DNA Extraction Kit, Sacace Biotechnologies Srl, Como, Italy) and used for an NGS analysis on a MiSeq Illumina instrument using a custom gene panel designed for cHH/KS (Supplementary Table S2). The target regions were enriched using an Illumina Nextera Rapid Capture Enrichment kit. The sequences were mapped on the human reference sequence GRCh38. Pathogenic variations were sought in the Human Gene Mutation Database (HGMD professional) and MASTERMIND (mastermind.genomenon). Sanger sequencing was used to confirm NGS variants and to also study variant segregation in family members.

Variants were filtered as follows: (1) variants with a minor allele frequency (MAF) lower than 1% in the 1000 Genomes (http://www.1000genomes.org/home (accessed on 1 September 2022)), EVS (http://evs.gs.washington.edu/EVS/ (accessed on 1 September 2022)), and GNOMAD (https://gnomad.broadinstitute.org/ (accessed on 1 September 2022)) databases were considered; (2) an evaluation focused on coding exons along with flanking ±15 intronic bases was carried out; and (3) the presence of synonymous and splicing variants with GMAF/MAX MAF inferior to the known frequency of the disease was verified on databases, such as The Human Gene Mutation Database (HGMD).

The interpretation of the variants was carried out using the scoring system of the American College of Medical Genetics and Genomics (ACMG) guidelines [21], with the help of the online tool VARSOME (https://varsome.com/ (accessed on 1 September 2022)) [22]. All variants related to the patient’s phenotype, except for the benign or likely benign ones, were reported. The highlighted variants were classified as pathogenic, likely pathogenic, and uncertain significance (VUS). MutationTaster (https://www.mutationtaster.org/ (accessed on 1 September 2022)) was used to evaluate the evolutionary conservation for missense variants.

### 2.3. Conservation of Protein Sequences among Species

To evaluate the conservation of amino acids across species, two types of entropies were computed: one was the Shannon entropy, which considers a uniform background distribution (H), and the second considers the BLOSUM62 background distribution (Hb) (i.e., taking into account the evolutionary preferences of specific amino acids) [23]. Multiple sequence alignment (MSA) was achieved by first using BLAST [24] to collect 100 evolutionary relatives from the non-redundant database and then using the MUSCLE server [25] to align the sequences with each other. The positions with gaps occupying more than 50% of the sequences were not considered in the calculations.

Entropy scores that do not incorporate background amino acid frequencies have been shown to be theoretically not optimal for calculating residue conservation. Therefore, we used another measure, called relative entropy or Kullback–Leibler divergence, which, in statistics, is used to show the distance between two probability distributions. In our case, it meant that the greater the deviation from the background distribution, the stronger the evolutionary constraint, implying the importance of a position in the alignment. As described in the previous section, since amino acids in a protein sequence are not sampled from a uniform distribution, but instead, their selection is driven by evolutionary forces, using a background frequency from sequence databases would produce more accurate results [26]. As suggested by different conservation measures [27], the BLOSUM62 background distribution was used.

### 2.4. Molecular Modeling

The mCSM web server was used [28] to evaluate the stabilization/destabilization effect of genetic variants on the protein structure. Homology modeling and threading were carried out using SWISS-Model to determine the structure of the proteins that were not experimentally solved, namely, FGF17, with PDB entry 2fdb as a template, and CHD7, with PDB entry 3mwy as a template.

Molecular dynamics simulations were carried out on both the wild-types and mutants of DUSP6, FGF17, and CHD7 using GROMACS version 2019.3 [29]. Each protein molecule was placed in a triclinic box with a minimum spacing of 1.2 nm on each face. The system was then solvated using TIP3P water molecules, neutralized with Na+/Cl−, and energy-minimized via the gradient descent algorithm (Fmax = 100 kJ mol^−1^ nm^−1^, nsteps = 50,000, step_size = 0.001). The minimized systems were subjected to two subsequential equilibration steps of position-restrained molecular dynamics in the NVT and NPT ensembles, 100 ps each. A reference temperature of 300 K and a pressure of 1 bar were imposed, respectively. Finally, a molecular dynamics production run was performed for 100 ns with a 2 fs integration step size in both wild-types and mutants.

A molecular dynamics analysis was performed by first calculating the Root-Mean-Square Deviation (RMSD) of the backbone atoms relative to the structure in the minimized and equilibrated system. Highly flexible inter-domain loops and terms were excluded from the RMSD due to their large movements during the simulation. The RMSD computation was then followed by Root-Mean-Square Fluctuations (RMSFs) to evaluate the fluctuations of the C-α atoms on a per-residue basis. To estimate the compactness of the structure during the simulation, the radius of gyration, the number of hydrogen bonds, and the extent of secondary structures were calculated.

## 3. Results

Using a customized panel of 31 genes (Supplementary Table S2), we found heterozygous variants in the *IL17RD*, *FGF17*, *DUSP6*, *PROK2*, *CHD7*, *FLRT3*, and *CCDC103* genes, 4 of which have never been reported in the literature. Four variants were classified as pathogenic, and five were classified as VUSs. Except for one, all patients carried at least two genetic variants.

Molecular modeling, molecular dynamics, and conservation analyses were possible for three of the nine variants identified in our patients, namely, the *FGF17* p.(Gly70Arg) found in Patient 1, the *DUSP6* p.(Leu145Arg) found in Patient 2, and the *CHD7* p.(Thr917Met) found in Patient 4. Monoallelic variants in autosomal recessive genes and nonsense variants were excluded. The FLRT3 protein was also excluded because the mutation p.(Ala369Val) is located in an inter-domain linker location with low prediction confidence.

In the conservation analysis, although the Shannon entropy scores were low (indicating functional/structural importance), the relative entropy scores showed that the amino acid distribution of the positions where the mutations were found was not significantly different from the background distribution. This indicates that the regions where our variants are located do not necessarily correspond to vital parts of the proteins, contrary to the implications of the Shannon entropy scores. However, entropy scores themselves are not definitive predictors of the functional or structural importance of a residue, and even if a position is poorly conserved, it does not imply that it can accommodate any amino acid, as was the case for the Leu145Arg variant in DUSP6.

The patients’ clinical phenotypes and genetic results are shown in Table 1 and Table 2.

### 3.1. Patient 1

Patient 1 was diagnosed with KS at the age of 18. Accordingly, he required testosterone replacement therapy (TRT) to reach full pubertal development. His pituitary MRI was unremarkable but showed the absence of the olfactory bulbs, which explained his anosmia. He searched for our counseling at the age of 42 years for infertility. He was given gonadotropin stimulation and could impregnate his wife using an assisted reproductive technique at the age of 43. Then, he was switched to TRT. Unfortunately, he died from pancreatic cancer at the age of 49. Family history was negative for cHH in all family members examined.

The genetic testing revealed the presence of the *IL17RD* c.960G>A, p.(Met320Ile) missense variant. The Met320 amino acid of the IL17RD protein is an evolutionarily conserved residue. The change with Ile was not predicted to alter the protein nature drastically; hence, the variant was interpreted as a VUS. He also carried the *FGF17* c.208G>A, p.(Gly70Arg) missense variant. Variants in this gene are known to be transmitted with an autosomal dominant (AD) mechanism.

**Figure 1:**
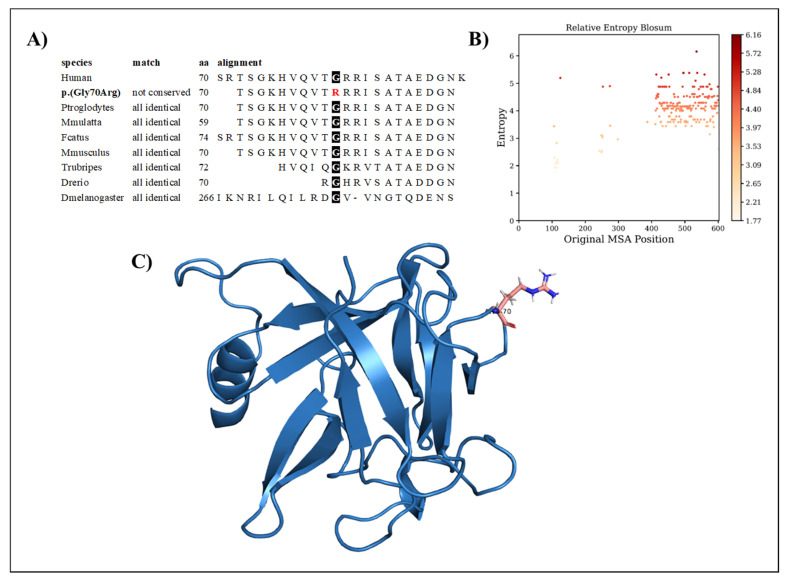
(**A**) Amino acid conservation across species of the FGF17 variant, showing that Gly70 is highly conserved. (**B**) Relative entropy plots. (**C**) The FGF17 Arg70 variant—highlighted in pink—resides in a loop and is oriented toward the solvent.

The variation regards a conserved amino acid position (Figure 1A) with normalized Shannon entropy scores of H = 0 and Hb = 0. The relative entropy, however, was among the lowest with a score of 3.59 (Figure 1B). To model *FGF17*, we used the structure provided by the Swiss model, based on the 2fdb.1.A template (Figure 1C). Positions 1 to 22 were discarded, as they are signal peptides, and the next 10 residues (positions 23 to 32) were missing from the Protein Data Bank (PDB) entry. Although the variant p.(Gly70Arg) replaces glycine with the more cumbersome arginine, it can be considered structurally neutral with a predicted stability change of ∆∆G = −0.324 kcal/mol because of its loop position, allowing for the charged sidechain to be in contact with the solvent.

### 3.2. Patient 2

Patient 2 is a 21-year-old female with secondary amenorrhea, poor development of sexual characteristics (Tanner 2 breasts), and osteopenia. She had spontaneous menstrual spotting at the age of 13, which was not followed by menses until the age of 16 when she did not respond to medroxyprogesterone acetate administration, and hormone replacement therapy was started. The MRI scan showed that she had no pituitary abnormalities, and no smell impairment was found. The hormonal evaluation revealed HH, with otherwise normal pituitary function. The patient was underweight (a body mass index of 15.3 Kg/m^2^), which supported the hypothetical diagnosis of functional hypogonadism. No family history of cHH was reported in first- and second-level siblings, except for palatoschisis, which was present in a cousin who did not have HH.

**Figure 2:**
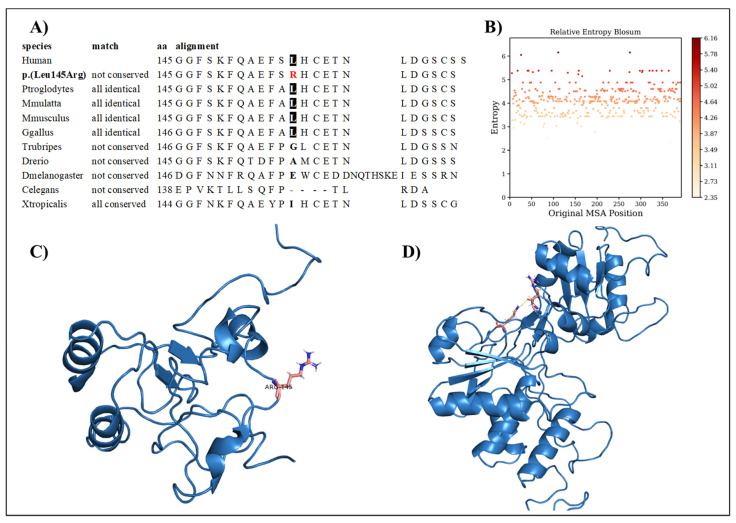
(**A**) Amino acid conservation across species of the DUSP6 variant, showing that Leu145 is moderately conserved. (**B**) Relative entropy plots. (**C**) Mutant DUSP6 with Leu145Arg highlighted in pink. (**D**) The DUSP6 AlphaFold model.

Interestingly, genetic testing showed the presence of the missense variant *DUSP6* c.434T>G, p.(Leu145Arg). The family segregation study showed that the proband inherited this variant from the father, who was healthy. The variation concerns a conserved amino acid position (Figure 2A) with normalized Shannon entropy scores of H = 0.01 and Hb = 0.02. The relative entropy was again among the lowest with a score of 3.36 (Figure 2B).

To simulate the dynamics of *DUSP6*, the structure of PDB entry 1HZM was used for molecular dynamics simulations of the Rhodanese domain. The variant replaces a hydrophobic amino acid (leucine) with a positively charged amino acid (arginine) (Figure 2C). The variant can be considered structurally neutral with a predicted stability change of ∆∆G: −0.07 kcal/mol.

### 3.3. Patient 3

Patient 3 is a 28-year-old male with cHH and CHARGE syndrome. Genetic testing showed two nonsense heterozygous variants, namely, *PROK2* c.163del, p.(Ile55*), predicted as pathogenic, and *CHD7* c.576T>A, p.(Tyr192), predicted as likely pathogenic. Family history was negative for cHH in all first- and second-level siblings investigated.

### 3.4. Patient 4

Patient 4 is a 28-year-old male with reversal cHH. The diagnosis was made at the age of 14 due to a small testicular volume and the absence of puberty. The hormone evaluation was compatible with HH. He started gonadotropin administration at the age of 14. At the age of 24, TRT was proposed, but, during the wash-out period, we found normal pituitary function and normal testosterone serum levels. Hence, the diagnosis of reversal cHH was made. Interestingly, at the genetic analysis, he had the *CHD7* c.2750C>T, p.(Thr917Met) and the *FLRT3* c.1106C>T, p.(Ala369Val) missense variants, both predicted as VUSs. The patient reported no history of cHH in any family member.

**Figure 3:**
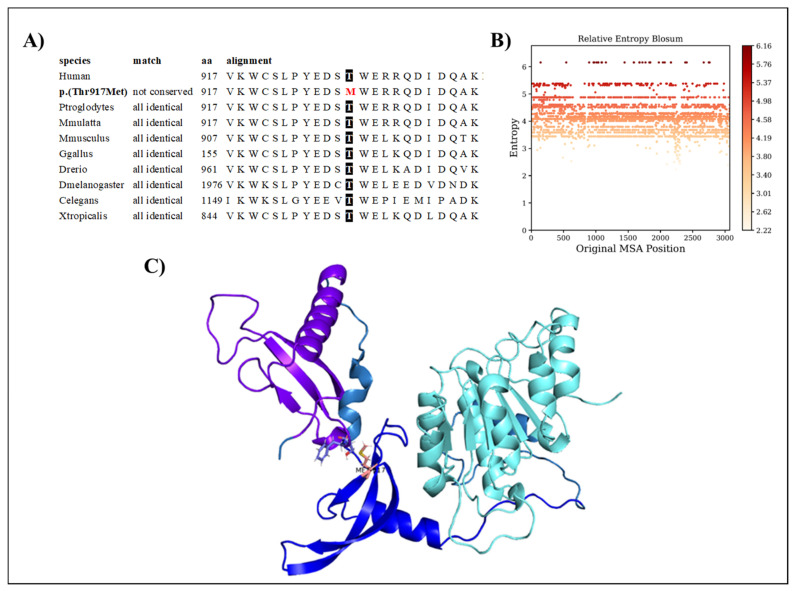
(**A**) Amino acid conservation across species of the CHD7 variant, showing that Thr917 is highly conserved. (**B**) Relative entropy plots. (**C**) CHD7 domains shown in different colors, with the variant shown in pink.

The CHD7 p.(Thr917Met) variant regards a conserved amino acid position (Figure 3A), with normalized Shannon entropy scores of H = 0 and Hb = 0. The relative entropy was still among the lowest with a score of 4.18 (Figure 3B).

To model CHD7, we used the structure provided by the Swiss model based on the 3mwy.1.A template. Only the chromo 1, chromo 2, and ATP-binding domains were prepared for the molecular dynamics simulation. Threonine at position 917 is faced toward a loop region extending the chromo1 domain (Figure 3C). The p.(Thr917Met) variant replaces it with methionine, which is highly hydrophobic and much larger. The variant is predicted to be structurally neutral with a predicted stability change of ∆∆G: −0.13 kcal/mol.

### 3.5. Patient 5

Patient 5 is a 27-year-old male with cHH and CHARGE syndrome. He started TRT at the age of 16; his smell and pituitary MRI scans were normal. The genetic testing revealed that he carried the *CHD7* c.7891C>T, p.(Arg2631*) nonsense variant and the *CCDC103* c.461A>C, p.(His154Pro) missense variants. Family history was negative for cHH in all family members examined.

### 3.6. Molecular Dynamics

#### 3.6.1. FGF17

In the FGF17 structure, the RMSF score difference between the wild-type and mutant, near the mutated region, is ~0.1 nm. Overall, the RMSF of both the wild-type and mutant fluctuates from ~0.05 nm to ~0.4 nm throughout the entire simulation, with a peak of ~0.42 nm at residues 40–55 in both structures, corresponding to a loop region. Residue 70 is positioned in a loop region connecting β2 and β3, which is in contact with the solvent. The replacement of G70 with the charged arginine slightly stabilizes the loop.

**Figure 4:**
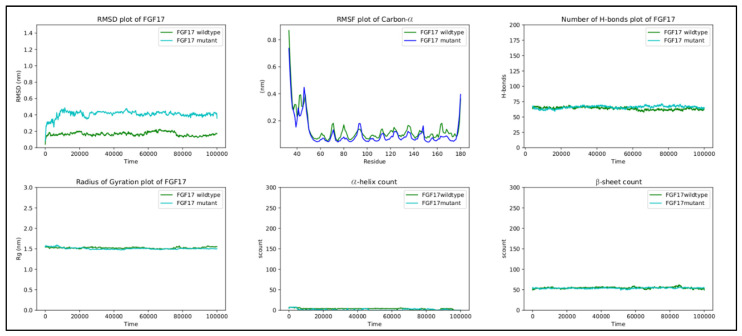
RMSD, RMSF, hydrogen bond count, radius of gyration, and secondary structure count plots of FGF17.

RMSD, the radius of gyration, the H-bond number, and the secondary structure number adhered to an invariant behavior throughout the simulation, providing evidence of the stability of the folded structure (Figure 4).

#### 3.6.2. DUSP6

**Figure 5:**
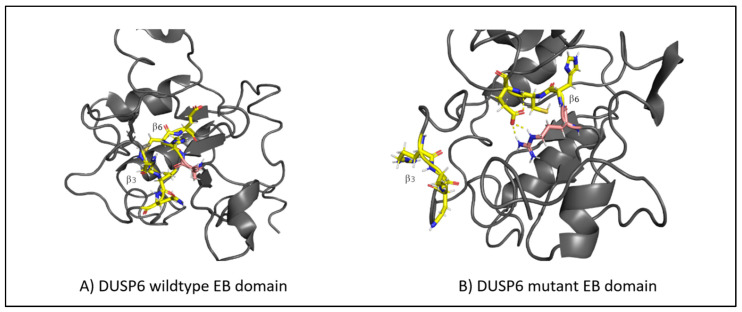
(**A**) The structure of the β-sheet (highlighted in yellow) in the wild-type DUSP6, with β3 tethering to β6. (**B**) The disruption of the β-sheet structure in the mutant DUSP6, with β3 untethering from β6. The mutation is highlighted in pink.

The RMSF values of the EB domain oscillate between ~0.1 nm and ~0.4 nm, excluding the termini. Two peaks are observed, reaching ~0.4 nm at regions between residues 20 and 40 and between residues 40 and 60. The first region corresponds to the loop connecting α1 to β2, and the second region corresponds to the loop connecting α2 to β3 [30]. For residues 56–89, comprising the β3-α3 region, the score remains reasonably low throughout the simulation, being in accordance with that in previous studies on the importance of this region to ERK2 binding [30]. However, the fluctuations of the mutant in the C-terminal are quite large. The C-terminal residues of the EB domain β6 145–147 form an anti-parallel β-sheet with the β3 65–67 residues (Figure 5A), both taking part in ERK2 binding [30]. Looking at the molecular dynamics’ trajectories, the mutant structure displayed the disruption of the β3-β6 sheet with β6 untethering from β3 (Figure 5B). Further molecular dynamic replicas of the wild-types and mutants supported this behavior, with the disruption occurring in 7 out of 11 mutant replicas compared to 3 out of 10 wild-type replicas. Moreover, in all mutant replicas, R65 was pushed back from its preferred conformation in the wild-type, possibly due to the introduction of R145. The importance of R65 in ERK2 binding has already been elucidated [30,31]. It may be concluded that the L145R variant disrupts the interaction between β6 and β3, needed for ERK2 binding and recognition.

**Figure 6:**
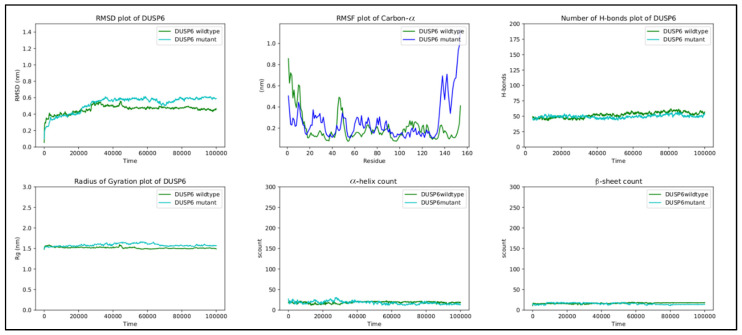
RMSD, RMSF, hydrogen bond count, radius of gyration, and secondary structure count plots of DUSP6.

RMSD, the radius of gyration, the H-bond number, and the secondary structure number adhered to an invariant behavior throughout the simulation, providing evidence of the stability of the folded structure (Figure 6).

#### 3.6.3. CHD7

**Figure 7:**
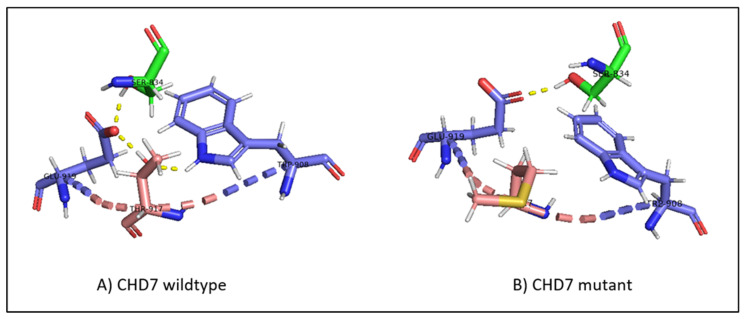
(**A**) The hydrogen bond network in wild-type CHD7, with T917 hydrogen bonding to E919 and W908, and E919 hydrogen bonding to S834. (**B**) The disruption of the hydrogen bond network in mutant CHD7. The introduction of the hydrophobic methionine causes the hydrogen bonds with E919 and W908 to be lost and M917 to flip away from the polar network. The mutation is highlighted in pink; the hydrogen bonds are highlighted in yellow dashed lines; residues belonging to the chromo 1 domain are highlighted in green.

In the wild-type *CHD*, threonine 917 is in proximity with serine 834 in the chromo 1 domain, tryptophan 908, and glutamate 919 in the chromo 2 domain. The hydrogen bonds between threonine and glutamate, threonine and tryptophan, and glutamate and serine form a hydrogen bond network that is present throughout the simulation (Figure 7A). The replacement of threonine by methionine at position 917 breaks the hydrogen bond network, with methionine flipping away due to its hydrophobic nature (Figure 7B).

Although RMSF oscillates in both structures, the differences in fluctuations between the wild-type and mutant are very small. The peaks observed between regions 810 and 825, 850 and 880, 890 and 905, 950 and 980, and 1050 and 1080 correspond to loop regions and inter-domain linkers.

**Figure 8:**
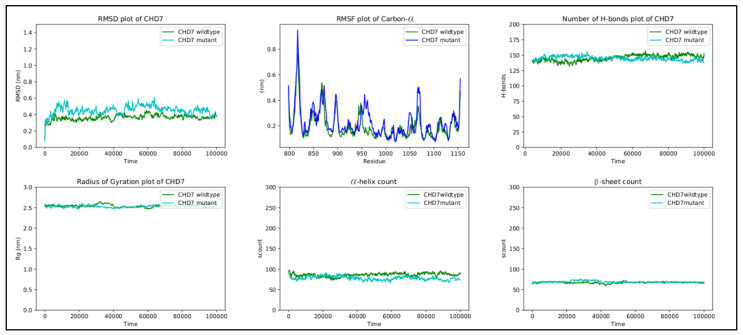
RMSD, RMSF, hydrogen bond count, radius of gyration, and secondary structure count plots of DUSP6 gene.

Finally, the other metrics provide evidence of the stability of the structure, with RMSD, the radius of gyration, the H-bond number, and the secondary structure count adhering to an invariant behavior throughout the simulation (Figure 8).

## 4. Discussion

CHH/KS is a rare clinical condition with a complex genetic etiology. Oligogenic transmission has apparently been reported in 2.5–15% of all patients with cHH [20]. NGS allows us to screen for a wide panel of genes in patients with cHH/KS and, thus, helps to identify new genes involved in the pathogenesis of this disease [32]. In detail, while we found previously reported heterozygous variants for the genes *PROK2* (c.163del, p.Ile55*), *CHD7* (c.c.2750C>T, p.Thr917Met and c.7891C>T, p.Arg2631*), *FLRT3* (c.1106C>T, p.Ala369Val), and *CCDC103* (c.461A>C, p.His154Pro), we also report a new pathogenic variant in the *CHD7* gene (c.576T>A, p.Tyr1928) and three new VUSs in heterozygosity for the *IL17RD* (c.960G>A, p.Met320Ile), *FGF17* (c.208G>A, p.Gly70Arg), and *DUSP6* (c.434T>G, p.Leu145Arg) genes.

Molecular modeling was applied to three VUSs, namely, the genes *IL17RD* (c.960G>A, p.Met320Ile), *FGF17* (c.208G>A, p.Gly70Arg), and *DUSP6* (c.434T>G, p.Leu145Arg), to understand their role in the pathogenesis of cHH. Although no possibility was predicted in the protein structure for the *IL17RD* and for the *FGF17* gene variants, the DUSP6 L145R variant was predicted to disrupt the interaction between β6 and β3, which is required for ERK2 binding and recognition. Thus, this variant could more likely play a role in the pathogenesis of cHH. Overall, these results suggest incomplete penetrance or variable expressivity as possible mechanisms of the cHH inheritance of the patients enrolled.

Variants in the *IL17RD* gene cause cHH with different phenotypic characteristics and transmission mechanisms (hypogonadotropic hypogonadism 18 with or without anosmia, OMIM 606807, associated with AD, AR, and DD transmission). Patients with cHH and heterozygous variants of this gene have been reported [33]. However, some authors have also found the concomitant presence of variants in other genes (*CHD7*, *ANOS1*, *FGFR1*, *LEPR*, *WDR11*, and *HS6ST1*) [34], thus indicating oligogenic inheritance. Therefore, heterogeneous loss-of-function IL17RD mutations may not be sufficient to cause the phenotype.

The *FGF17* gene maps in the chromosome 8p2.3 and shares a high homology (61%) with the *FGF8* gene, coding for an important protein highly expressed in the medial olfactory placode from where GnRH neurons originate [35]. Miraoui and colleagues identified three patients with cHH (one female and two males) who carried heterozygous *FGF17* variants. The female patient also carried *FLRT3*, *HS6ST1*, and *FGFR1* variants and showed a reduced bone mass. Interestingly, the two male patients carried only heterozygous variants in the *FGF17* gene, and only one of them had a reduced bone mass [36]. Finally, three new variants have recently been identified in three male patients with cHH, two of whom had olfactory bulb aplasia on MRI [34], similar to the case of Patient 1 in the present study.

The single contribution of these variants in the pathogenesis of cHH in Patient 1 is unknown. The *FGF17* gene variant could play a predominant role, although the contribution of the *IL17RD* gene variant on the Patient 1 KS phenotype cannot be excluded entirely.

Heterozygous variants in the *DUSP6* gene have been associated with patients with cHH in the literature [37,38], which increases its likelihood of playing an etiopathogenic role in the patient’s phenotype here described. Indeed, five patients carrying *DUSP6* variants were described by Miraoui and colleagues. Two females with KS, both with a reduced bone mass and one with hearing loss, carried digenic variants of the genes DUSP6 p.(Asn189Ser) and SPRY4, p.(Ser241Tyr). Another female patient carrying a monoallelic *DUSP6* variant p.(Phe77Ile) had normosmic hypogonadotropic hypogonadism and abnormal dentition.

The *DUSP6* gene is known to influence the migration of GnRH neurons, and its variants are transmitted with an AD mechanism. However, the family segregation study (considering the proband’s healthy parents and sister) showed that our proband inherited this variant from her father. The patient herself was suspected of having functional hypogonadism due to her low body mass index. On this basis, we hypothesized that (1) the *DUSP6* variant is associated with variants in other hypogonadotropic hypogonadism genes that were not evaluated in our proband, which is a possibility according to the literature [37]; (2) this gene may cause the disease via a mechanism of incomplete penetrance; and (3) the interaction between a “non-severe” gene variation and a specific acquired risk factor may lead to the development of the pathological phenotype. This raises the possibility that the onset of functional hypogonadotropic hypogonadism is influenced by the patient’s specific genetic background. Finally, it should be emphasized that molecular modeling revealed that the variant’s effect on the protein was quite dramatic; thus, we have a clear lead regarding its pathogenic role, which can be subjected to further studies.

Surprisingly, while this patient may appear not to have a congenital form of HH, her case allows us to discuss the presence of a possible genetic factor. Indeed, the presence of palatoschisis in a cousin makes the diagnosis of cHH not excludable since palatoschisis is one of the phenotypic features that can occur in patients with cHH. Once again, the presentation of this case highlights how the environment (low BMI) and mild genetic factors (gene variant and positive family history) could influence the onset of the phenotype.

*CHD7* variants can cause CHARGE syndrome, whose major criteria are coloboma, choanal atresia, and hypoplasia of the semicircular canals, while rhomb encephalic dysfunction, ear anomalies, intellectual disability, hypothalamic–pituitary dysfunction, and malformation of mediastinal organs are considered minor diagnostic criteria.

Some studies have shown that this gene, when mutated, can cause cHH [34,36,37,38]. In particular, Kim and colleagues identified *CHD7* variants in ~6% of patients with cHH/KS without the presence of a significant CHARGE phenotype. In contrast, another study found that 80% of patients with HH with *CHD7* variants show several CHARGE features [12]. In line with the literature, Patient 4 carried pathogenic variants in two different genes, *CHD7* and *PROK2*. The nonsense *CHD7* has never been reported in the literature. It could explain the CHARGE phenotype observed in this patient, while the *PROK2* variant has already been described in the literature in association with cHH/KS [16,39], and its pathogenicity was supported by functional studies [1].

One interesting insight that can be inferred from our case series is that of Patient 4. He carried heterozygous variants of the *CHD7* and *FLRT3* genes and underwent cHH reversal. *FLRT3* could be implicated in cHH/KS pathogenesis, as previously reported [37,40].

The *FLRT3* variant found in Patient 4 has already been described in the literature as a rare missense variant of unknown association with CHARGE syndrome [41]. The *CHD7* variant, already described by our group [42], is a VUS of difficult interpretation, but molecular modeling suggests a potential pathogenic role. If it were true, Patient 4 would be the first case of reversal cHH associated with a *CHD7* variant.

The p.(Arg2631*) CHD7 variant found in Patient 5 has been described as a recurrent variant in patients with CHARGE [43], thus representing alone the genetic cause of the phenotype observed in our patient. Indeed, the second variant found in the heterozygous state is not able to cause disease, as only one allele is affected (Table 2).

**Table 2:** Clinical and biochemical features of the patients with cHH.

Patient ID	Sex	Gene	Refseq	Variant	Type of Variant	Genotype	Inheritance	Zygosity	MAF	Familiar Segregation	Variant Classification	References
1	M	*IL17RD*	NM_017563.4	c.960G>A	Missense	p.(Met320Ile)	AD/AR/DD	Het	0.008%	N/A	VUS	Novel variant
		*FGF17*	NM_003867.3	c.208G>A	Missense	p.(Gly70Arg)	AD	Het	0.008%	N/A	VUS	Novel variant
2	F	*DUSP6*	NM_001946.3	c.434T>G	Missense	p.(Leu145Arg)	AD	Het	-	YES	VUS	Novel variant
3	M	*PROK2*	NM_001126128.1	c.163del	Nonsense	p.(Ile55*)	AD	Het	-	N/A	Pathogenic	[44]
		*CHD7*	NM_017780	c.576T>A	Nonsense	p.(Tyr192*)	AD	Het	-	N/A	Likely Pathogenic	Novel variant
4	M	*CHD7*	NM_017780.3	c.2750C>T	Missense	p.(Thr917Met)	AD	Het	-	N/A	VUS	[42]
		*FLRT3*	NM_198391.2	c.1106C>T	Missense	p.(Ala369Val)	AD	Het	0.0036%	N/A	VUS	[41]
5	M	*CHD7*	NM_017780.4	c.7891C>T	Nonsense	p.(Arg2631*)	AD	Het	-	N/A	Pathogenic	[41]
		*CCDC103*	NM_213607.3	c.461A>C	Missense	p.(His154Pro)	AR	Het	0.3234%	N/A	Pathogenic	[43]

Abbreviations. AD, autosomal dominant; AR, autosomal recessive; Het, heterozygous; cHH, congenital hypogonadotropic hypogonadism; F, female; M, male; VUS, variance of uncertain significance. CCDC103, coiled-coil domain-containing protein 103; CHD7, Chromodomain Helicase DNA Binding Protein 7; DUSP6, dual-specificity phosphatase 6; FGF17, Fibroblast Growth Factor 17; FLT3, FMS-related tyrosine kinase 3; IL17RD, Interleukin 17 Receptor D; PROK2, Prokineticin 2.

## 5. Limitations and Strengths

The case series presented in this study have some limitations. Firstly, the number of cases is low. However, most patients have cHH diagnosed according to the clinical, hormonal, and radiological criteria of the European Consensus Statement document [4]. Secondly, another reason why these data should be interpreted with caution is the absence of a functional analysis confirming the effects of the gene variants on protein function. Thirdly, the absence of genetic testing in the relatives of Patients 1, 3, 4, and 5 represents another why our conclusion should be taken with caution. Additionally, the filtering method of flanking ±15 intronic bases and MAF lower than 1% might also lead to the overlooking of some variants. However, the rigorous diagnostic criteria used to select the patients, the wide panel of genes used, and the validation of our results through molecular modeling represent the strengths of this study. Furthermore, molecular modeling, molecular dynamics, and conservation analyses were performed for three out of the five variants described as VUSs, for which there is no evidence in the literature, namely, *FGF17*, *DUSP6*, and *CHD7* p.(Thr917Met). This adds a new and original contribution to the field.

## 6. Conclusions

In conclusion, we found new genetic variants potentially involved in the pathogenesis of patients with cHH/KS. In detail, we found previously reported heterozygous variants for the genes *PROK2* (c.163del, p.Ile55*), *CHD7* (c.c.2750C>T, p.Thr917Met and c.7891C>T, p.Arg2631*), *FLRT3* (c.1106C>T, p.Ala369Val), and *CCDC103* (c.461A>C, p.His154Pro). More importantly, we report here one new pathogenic variant of the *CHD7* gene (c.576T>A, p.Tyr1928) and three new VUSs for the genes *IL17RD* (c.960G>A, p.Met320Ile), *FGF17* (c.208G>A, p.Gly70Arg), and *DUSP6* (c.434T>G, p.Leu145Arg) occurring in the heterozygous state. Interestingly, the molecular modeling results suggest the role of *DUSP6* in the pathogenesis of cHH in Patient 2. Consequently, the L145R variant disrupts the interaction between β6 and β3, required for ERK2 binding and recognition. Conversely, our analysis indicates that it is unlikely that the VUSs of *IL17RD* (c.960G>A, p.Met320Ile) and *FGF17* (c.208G>A, p.Gly70Arg) are involved in the pathogenesis of cHH. Functional studies are needed to confirm the role of *DUSP6* (c.434T>G, p.Leu145Arg) in the pathogenesis of cHH.

Furthermore, the present case series offer interesting insights into the relationship between the genotype and the environmental risk factors in the onset of functional hypogonadotropic hypogonadism and the genetic causes of reversal cHH. As NGS is increasingly used in the genetic diagnosis of patients with cHH/KS, we hypothesize that the mechanisms of inheritance will be increasingly elucidated.

## Figures and Tables

**Table 1:** Clinical and biochemical features of the patients with congenital hypogonadotropic hypogonadism (cHH)/Kallmann syndrome (KS).

	Patient 1 ♂	Patient 2 ♀	Patient 3 ♂	Patient 4 ♂	Patient 5 ♂
Current age	Died at age 49 from pancreatic cancer	21 years	28 years	28 years	27 years
Phenotype	KS	Functional hypogonadism	cHH	cHH	cHH
Overlapping syndromes	No	No	CHARGE	No	CHARGE
GnRH reversal	No	No	No	Yes	No
Gene(s)	*IL17RD*/*FGF17*	*DUSP6*	*PROK2*/*CHD7*	*CHD7*/*FLRT3*	*CHD7*/*CCDC103*
Genotype	c.960G>A, p.(Met320Ile) in heterozygosis/c.208G>A, p.(Gly70Arg) in heterozygosis	c.434T>G, p.(Leu145Arg) in heterozygosis	c.163del, p.(Ile55*) in heterozygosis/ c.576T>A, p.(Tyr192*) in heterozygosis	c.2750C>T, p.(Thr917Met) in heterozygosis/c.1106C>T, p.(Ala369Val) in heterozygosis	c.7891C>T, p.(Arg2631*), in heterozygosis/ c.461A>C, p.(His154Pro) in heterozygosis
Smell	Anosmia	Normal	Normal	Normal	Normal
Testicular volume (mL) * or primary amenorrhea	6	No	1	5	3
Total testosterone (ng/dL) or 17ß-estradiol (pg/mL)	11	<10	49	78	0.6
Luteinizing hormone (IU/L)	0.3	0.01	0.07	3.9	0.1
Follicle-stimulating hormone (IU/L)	0.9	0.06	0.3	2.59	0.1
Magnetic resonance imaging	Atrophy of the olfactory bulb	Normal	Empty Sella	Normal	Normal

Abbreviations: CHARGE: coloboma, heart defect, atresia choanae, retarded growth and development, genital hypoplasia, ear anomalies/deafness syndrome. CCDC103, coiled-coil domain-containing protein 103; CHD7, Chromodomain Helicase DNA Binding Protein 7; DUSP6, dual-specificity phosphatase 6; FGF17, Fibroblast Growth Factor 17; FLT3, FMS-related tyrosine kinase 3; IL17RD, Interleukin 17 Receptor D; PROK2, Prokineticin 2. * Mean volume using ultrasound scan. Normal value of total testosterone: 300–1000 ng/dL; normal value of 17ß-estradiol: 30–400 pg/mL; normal value of luteinizing hormone: 1.5 to 12.4 IU/L; normal value of follicle-stimulating hormone: 1.5 to 12.4 IU/L.

## Data Availability

Data will be made available upon request to the corresponding author.

## Supplementary Materials

The following supporting information can be downloaded at https://www.mdpi.com/article/10.3390/ijms24087428/s1.
