# Supplementary material for: Genetic Analysis of Patients with Congenital Hypogonadotropic Hypogonadism: A Case Series
Source: Int J Mol Sci. 2023 Apr 18;24(8):7428. doi: 10.3390/ijms24087428 (PMC10138801; doi:10.3390/ijms24087428)
Supplement: Supplementary file 1 [file ijms-24-07428-s001.zip › ijms-2341235-supplementary.pdf]

International Journal of
Molecular Sciences

Supplementary Table S1. Genes involved in the pathogenesis of congenital hypogonadotropic hypogonadism [5].

Function	Genes
GnRH neuron differentiation	*FGF8/FGFR1, SOX2, CHD7, FGF17, IL17RD*
GnRH neuron migration and axon projection	*ANOS1, PROK2/PROKR2, SEMA3A/PLXNA1, SEMA3E, NSMF, HS6ST1, WDR11, SOX10, FEZF1 IGSF10, DCC/NTN1, TUBB3, SMCHD1*
GnRH neuron homeostasis	*GNRH1, KISS1/KISS1R, TAC3/TACR3, LEP/LEPR, PCSK1, DXML2, KLB*
Defects in gonadotropic cells	*GNRHR, NR0B1, PNOLA6, POLR3B*
Unclear	*OTUD4, RNF216*

Abbreviations. *ANOS1, Anosmin 1; CHD7, Chromodomain Helicase DNA Binding Protein 7; DCC, Deleted in Colorectal Carcinoma; DXML2, DMX-like 2; FEZF1, Fez family zinc finger protein 1; FGF8, Fibroblast Growth Factor 8; FGF17, Fibroblast Growth Factor 17; FGFR1, Fibroblast growth factor receptor 1; GNRH1, Gonadotropin Releasing Hormone 1; GNRHR, Gonadotropin Releasing Hormone Receptor; HS6ST1, Heparan Sulfate 6-O-Sulfotransferase 1; IGSF10, Immunoglobulin superfamily member 10; IL17RD, Interleukin 17 Receptor D; KLB , Klotho Beta; LEP, leptin; LEPR, leptin receptor; KISS1, kisspeptin 1; KISS1R, kisspeptin 1 receptor; NR0B1, Nuclear Receptor Subfamily 0 Group B Member 1; NSMF, NMDA receptor synaptonuclear signaling and neuronal migration factor; NTN1, netrin 1; OTUD4, OTU domain-containing protein 4; PCSK1, Proprotein Convertase Subtilisin/Kexin Type 1; PLXNA1, plexin a1; PNOLA6, Patatin-like phospholipase domain-containing protein 6; POLR3B, Polymerase III, RNA, subunit B; PROK2, Prokineticin 2; PROKR2, Prokineticin receptor 2; RNF216, Ring finger protein 216; SEMA3A, Semaphorin 3A; SEMA3E, Semaphorin 3E; SMCHD1, Structural Maintenance of Chromosomes flexible Hinge Domain Containing 1; SOX2, Sex determining region Y-box 2; SOX10, Sex determining region Y-box 10; TAC3, Tachykinin 3; TACR3, Tachykinin receptor 3; TUBB3, Tubulin Beta 3 Class III; WDR11, WD repeat-containing protein 11.*

Supplementary Table S2. Customized gene panel for congenital hypogonadotropic hypogonadism/Kallmann syndrome.

Gene	Gene MIM number	Inheritance	Chromosomal locus	OMIM Phenotype	HH cases due to the gene variants*
KISS1	603286	AR	1q32.1	HH, 13 with or without anosmia	<2% (nHH)
HS6ST1	604846	AD	2q14.3	HH, 15 with or without anosmia	<2% (KS or nHH)
IL17RD	606807	AD	3p14.3	HH, 18 with or without anosmia	2-5% (KS or nHH)
PROK2	607002	AD	3p13	HH, 4 with or without anosmia	<2% (KS or nHH)
GNRHR	138850	AR	4q13.2	HH, 7 with or without anosmia	5-10% (nHH)
TACR3	162332	AR	4q24	HH, 11 with or without anosmia	~5% (nHH)
SPRY4	607984	AD	5q31.3	HH, 17 with or without anosmia	<2% (KS or nHH)
SEMA3A	603961	AD	7q21.11	HH, 16 with or without anosmia	<2% (KS or nHH)
FEZF1	613301	AR	7q31.32	HH, with or without anosmia	<2% (KS)
FGF17	603725	AD	8p21.3	HH, 20 with or without anosmia	<2% (KS or nHH)
GNRH1	152760	AR	8p21.2	HH, 12 with or without anosmia	<2% (nHH)
FGFR1	136350	AD	8p11.23	HH, 2 with or without anosmia	~10% (KS or nHH)
CHD7	608892	AD	8q12.2	HH, 5 with or without anosmia	5-10% (KS or nHH)
NSMF	608137	AD	9q34.3	HH, 9 with or without anosmia	---
FGF8	600483	AD	10q24.32	HH, 6 with or without anosmia	<2% (KS or nHH)
WDR11	606417	AD	10q26.12	HH, 14 with or without anosmia	<2% (KS or nHH)
FSHB	136530	AR	11p14.1	HH, 24 with or without anosmia	---
TAC3	162330	AR	12q13.3	HH, 10 with or without anosmia	<2% (nHH)
DUSP6	602748	AD	12q21.33	HH, 19 with or without anosmia	<2% (KS or nHH)
KISS1R	604161	AR	19p13.3	HH, 8 with or without anosmia	<2% (nHH)
LHB	152780	AR	19q13.33	HH, 23 with or without anosmia	
PROKR2	607123	AD	20p12.3	HH, 3 with or without anosmia	~5% (KS or nHH)
FLT3	604808	AD	20p12.1	HH, 21 with or without anosmia	<2% (KS or nHH)
ANOS1 (KAL1)	300836	XLR	Xp22.31	HH, 1 with or without anosmia (Kallmann syndrome 1, KS)	5-10% (KS)
SOHLH1	610224	AR	9q34.3	Hypergonadotropic hypogonadism, nonsyndromic	---
SOX10	602229	AD	22q13.1	Kallmann syndrome, with or without deafness	2-5% (KS)
AXL	109135	AD	19q13.2	HH	<2% (KS or nHH)
CCDC141	616031	AR	2q31.2	KS	<2% (KS)

SEMA3E	608166	AD	7q21.11	KS	<2% (KS or nHH)
SRA1	603819	AR	5q31.3	HH	<2% (nHH)

Abbreviations: *AD, autosomal dominant; AR, autosomal recessive; HH, hypogonadotropic hypogonadism; KS, Kallmann syndrome; nHH, normosmic hypogonadotropic hypogonadism. ANOS1, Anosmin 1; AXL, AXL receptor tyrosine kinase; CCDC141, Coiled-coil domain-containing protein 141; CHD7, Chromodomain Helicase DNA Binding Protein 7; DUSP6, Dual-specificity phosphatase 6; FSHB, Follicle-stimulating hormone, beta polypeptide; FEZF1, Fez family zinc finger protein 1; FGF8, Fibroblast Growth Factor 8; FGF17, Fibroblast Growth Factor 17; FGFR1, Fibroblast growth factor receptor 1; FLT3, FMS-related tyrosine kinase 3; GNRH1, Gonadotropin Releasing Hormone 1; GNRHR, Gonadotropin Releasing Hormone Receptor; HS6ST1, Heparan Sulfate 6-O-Sulfotransferase 1; IL17RD, Interleukin 17 Receptor D; LHB, luteinizing hormone, beta polypeptide; KISS1, kisspeptin 1; KISS1R, kisspeptin 1 receptor; NSMF, NMDA receptor synaptonuclear signaling and neuronal migration factor; PROK2, Prokineticin 2; PROKR2, Prokineticin receptor 2; SEMA3A, Semaphorin 3A; SEMA3E, Semaphorin 3E; SOHLH1, Spermatogenesis- and oogenesis-specific basic helix-loop-helix protein 1; SOX10, Sex determining region Y-box 10; SPRY4, Sprouty RTK signaling antagonist 4; SRA1, Steroid receptor RNA activator 1; TAC3, Tachykinin 3; TACR3, Tachykinin Receptor 3; WDR11, WD repeat-containing protein 11.*

**GeneReview, https://www.ncbi.nlm.nih.gov/books/NBK1334/, Rev. March, 2017.*
